# Detection of *Salmonella* Typhimurium on Spinach Using Phage-Based Magnetoelastic Biosensors

**DOI:** 10.3390/s17020386

**Published:** 2017-02-16

**Authors:** Fengen Wang, Shin Horikawa, Jiajia Hu, Howard C. Wikle, I-Hsuan Chen, Songtao Du, Yuzhe Liu, Bryan A. Chin

**Affiliations:** 1 Institute of Quality Standard and Testing Technology for Agro-Products, Shandong Academy of Agricultural Sciences, Jinan 250100, China; 2 Materials Research and Education Center, Auburn University, Auburn, AL 36849, USA; horiksh@auburn.edu (S.H.); hcw0002@auburn.edu (H.C.W.III); SZD0032@auburn.edu (S.D.); yzl0124@auburn.edu (Y.L.); chinbry@auburn.edu (B.A.C.); 3 Jiangsu Key Laboratory of Materials Surface Technology, School of Materials Science and Engineering, Changzhou University, Changzhou 213164, China; hujiajia242187@126.com; 4 Department of Biological Sciences, Auburn University, Auburn, AL 36849, USA; chenih1@auburn.edu

**Keywords:** *Salmonella* Typhimurium, spinach, magnetoelastic, biosensors

## Abstract

Phage-based magnetoelastic (ME) biosensors have been studied as an in-situ, real-time, wireless, direct detection method of foodborne pathogens in recent years. This paper investigates an ME biosensor method for the detection of *Salmonella* Typhimurium on fresh spinach leaves. A procedure to obtain a concentrated suspension of *Salmonella* from contaminated spinach leaves is described that is based on methods outlined in the U.S. FDA Bacteriological Analytical Manual for the detection of *Salmonella* on leafy green vegetables. The effects of an alternative pre-enrichment broth (LB broth vs. lactose broth), incubation time on the detection performance and negative control were investigated. In addition, different blocking agents (BSA, Casein, and Superblock) were evaluated to minimize the effect of nonspecific binding. None of the blocking agents was found to be superior to the others, or even better than none. Unblocked ME biosensors were placed directly in a concentrated suspension and allowed to bind with Salmonella cells for 30 min before measuring the resonant frequency using a surface-scanning coil detector. It was found that 7 h incubation at 37 °C in LB broth was necessary to detect an initial spike of 100 cfu/25 g *S.* Typhimurium on spinach leaves with a confidence level of difference greater than 95% (*p* < 0.05). Thus, the ME biosensor method, on both partly and fully detection, was demonstrated to be a robust and competitive method for foodborne pathogens on fresh products.

## 1. Introduction

The increased awareness of the benefits of a healthy diet has led to an increase in the consumption of fresh fruits and vegetables [1,2] along with a concomitant increase in the number of foodborne illnesses [1,3,4]. Recent examples of outbreaks related to fruits and vegetables include cases of infection with *Escherichia coli O157:H7*, *Campylobacter jejuni*, *Listeria monocytogenes*, *Shigella spp.*, *Staphylococcus aureus* and *Salmonella spp*. [1,5,6]. Among these infectious agents, *Salmonella* has been associated with fresh produce including tomatoes, seed sprouts, cantaloupe, mamey, apple juice, orange juice, and spinach [7]. Since contamination may occur at any stage during production, packaging, transportation or storage, it is essential to develop rapid, sensitive, specific and inexpensive detection technologies for the identification of contaminated produce [8].

Phage-based magnetoelastic (ME) biosensors have been investigated as a candidate for in-situ, real-time, wireless, direct detection method for foodborne pathogens [8,9,10,11,12,13]. An ME biosensor is composed of a free-standing ME resonator serving as the signal transducer that is coated with a genetically engineered phage serving as the biomolecular-recognition element. The detection principle is based on measuring the change in the sensor’s resonant frequency, which is proportional to the change in the sensor’s mass [14]. Due to the Joule magnetostriction effect, the biosensor can be placed into mechanical resonance when subjected to a time-varying magnetic field oscillating at the proper frequency. When pathogens are captured by the phage coating, the increase in the biosensor mass causes a decrease in its resonant frequency. The ME biosensors have been used successfully for pathogen detection such as *Salmonella*, *Bacillus anthracis spores* and *E. coli*. In previous work, the ME biosensors were placed directly on food surfaces for *Salmonella* Typhimurium detection [15,16], or real-time monitoring in various processes [17,18]. Compared with polymerase chain reaction (PCR), the gold standard for the detection of pathogens in foods [8], ME biosensors performed cost and time effective, simple to operate and LOD competitive [19,20]. Given these advantages, ME biosensor method was considered to be a robust and promising alternative to the Q-PCR method. However, in previous work, the samples were only partly detected, which may result in less representativeness and repeatability. To reinforce and develop the practicability and applicability of the ME biosensor method, full detection assessment should be considered and developed.

This paper investigates a ME biosensor method for the detection of *Salmonella* Typhimurium on fresh spinach leaves based on the isolation of *Salmonella* from leafy green vegetables outlined in the U.S. FDA Bacteriological Analytical Manual. The whole sample contributed to the detection and the signal was amplified by bacteria pre-enrichment. The effects of an alternative pre-enrichment broth (LB broth vs. lactose broth), incubation time on the detection performance and negative control were investigated. In addition, different blocking agents (BSA, Casein, and Superblock) were compared to minimize the effect of nonspecific binding on the ME biosensor surfaces.

## 2. Materials and Methods

In the present work, bare ME sensor platforms are defined as gold-coated strips of the ME alloy. Measurement biosensors are ME sensor platforms that have E2 phage immobilized onto the surface and blocked with a blocking agent. Control biosensors are prepared identically to measurement sensors except that they are devoid of phage. Control biosensors are used to compensate for environmental effects and non-specific binding. 

### 2.1. Fabrication of ME Biosensor Platforms

A ribbon of METGLAS 2826MB alloy was diced into strips (1 mm × 0.2 mm × 0.028 mm) using an automated dicing saw. The METGLAS strips were ultrasonically cleaned in acetone followed by methanol, then air-dried. The cleaned strips were annealed at 220 °C in vacuum (10^−3^ Torr) for 2 h to relieve residual internal stresses from the dicing process. Two metal layers (Cr followed by Au) were then sputter-deposited onto the strip surfaces to form the biosensor platform. The Cr layer acts as an adhesive interface between the strip and the Au layer. The Au layer not only protects the METGLAS strip from corrosion but also provides a ready surface for bio-probe immobilization. Finally, the bare biosensor platforms were annealed again to correct possible surface defects that might have occurred during the metal deposition process.

### 2.2. E2 Phage Immobilization

Filamentous E2 phage has been studied and verified to be highly specific and selective towards *S.* Typhimurium [21,22]. E2 phage with a concentration of 5 × 10^11^ vir/mL in a 1X tris-buffered saline (TBS) solution was prepared and provided by Dr. James M. Barbaree's lab in the Department of Biological Sciences at Auburn University, Auburn, AL. The bare ME biosensor platforms were immersed in the phage solution and rotated for 1 h. In this way, the phage attached uniformly to the platforms via physical adsorption. These biosensors with immobilized phage were then washed with deionized water twice to remove any unbound/loosely bound phage and salt originating from the TBS solution. 

### 2.3. Surface Blocking of the ME Biosensors

Three blocking reagents (BSA, casein, and Superblock) were evaluated in an effort to minimize non-specific binding of *Salmonella* to the biosensors. Bare ME biosensor platforms were either coated with one of the surface blockers or left uncoated. The ME biosensor platforms, were exposed for 30 min to a *S.* Typhimurium solution (5 × 10^8^ cfu/mL) in a PCR tube with rotation. Frequency shift measurements and optical microscopy, of 5 parallel biosensors per blocker, were used to compare the performance of the surface blockers.

### 2.4. Salmonella Pre-Enrichment and Exposure to the Biosensors

*Salmonella enterica* serovar Typhmurium (ATCC 13311) was provided by Dr. James. M. Barbaree’s lab in the Department of Biological Sciences at Auburn University, Auburn, AL. Fresh spinach leaves, from local grocery and sterilized by alcohol, were collected into a sterile plastic bag and spiked with *S.* Typhimurium (100 cfu/25 g spinach). The spiked leaves were removed from the bag, mixed on aluminum foil for 10 min to homogenize the mixture and returned to the bag. The spiked leaves were allowed to rest for 1 h at 4 °C.

The pre-enrichment and centrifugation procedure generally followed U.S. FDA’s Bacteriological Analytical Manual (BAM) [23]. Lactose broth, specified in BAM as the pre-enrichment broth for leafy green vegetables and herbs, and LB-Lennox broth were compared in this work. The 25 g sample of the spiked spinach leaves were aseptically transferred into 50 mL pre-enrichment broth inside a sterile bag within a beaker. The sterile bag was folded loosely to ensure oxygen aeration for bacteria growth. All samples were held at room temperature for 1 h and then incubated at 37 °C for up to 22 h.

Aliquots (1.5 mL) of the pre-enrichment broth were collected at 5, 6, 7, 14, and 22 h and centrifuged at 10,000 rpm for 10 min. The supernatant was replaced with 300 μL filtered water, and *Salmonella* cells were re-suspended by overtaxing. The *Salmonella* suspension was then transferred to PCR tubes containing a ME biosensor and vibrated for 30 min. The biosensors were finally collected, washed with deionized water twice and air-dried in preparation for resonant frequency measurements and optical microscopy.

The performance of Lactose broth and LB-Lennox broth was compared using optical microscopy. 10 bare ME biosensor platforms for both broth were prepared in *Salmonella* suspension obtained after spinach leaves were incubated at 5 h, and the micrographs of platform surfaces indicates the better broth for incubation.

### 2.5. Frequency Measurement and Student’s t-Test

The resonant frequency of the biosensors, of 10 parallel sensors for suspensions derived from each incubation time in LB broth, was measured with a surface-scanning coil detector, and a network analyzer with an S-parameter test set [17]. By comparing the initial and final resonant frequencies of the biosensors, measured before and after exposure to *Salmonella*, taking a mean value after 5 tests, the frequency shifts can be calculated. The frequency shifts are proportional to the mass changes on the sensor surfaces, allowing quantification of the amount of attached *S.* Typhimurium cells.

Finally, a one-tail unpaired Student’s *t*-test was performed to analyze the degree of dissimilarity between the measurement and control sensors in order to determine the minimum incubation time required for detection.

### 2.6. Optical Microscopy Observation

Confirmation of phage immobilization was conducted by fluorescent microscopy. E2 phage was labeled with the Alexa Fluor 488 dye in advance, following the procedure previously described [21]. Fluorescence micrographs of the phage-coated and bare ME biosensor surfaces were captured with an Olympus BX51 microscope equipped with a Lumen 200 metal halide fluorescence light source and a fluorescein isothiocyanate (FITC) filter set. 40X objective was used with a 100% light output intensity, an exposure time of 10.2 s and a gain of 16. The GNU Image Manipulation Program (GIMP) was used for image processing, including gray-scale conversion, background subtraction and thresholding, to calculate the surface phage coverage. 

*Salmonella* binding was also observed and confirmed by optical microscopy. Following exposure to *S.* Typhimurium, the biosensors were washed with distilled water and exposed to an osmium tetroxide (OsO_4_) vapor for 40 min for fixation of the bacteria cells. The washing procedure helps to remove unbound or weakly bound bacteria cells and also cleans the debris (such as salts) from the sensor surface. The biosensors were then exposed to a dye (crystal violet, 5% in distilled water) for 10 min, washed with distilled water and dried. Optical microscopy (Olympus BX51, Olympus Optical, Tokyo, Japan) was performed to observe the binding of *S.* Typhimurium on the sensor surfaces.

### 2.7. Negative Control Test

*Bacillus anthracis spores* and *E. coli* worked as the masking bacterial cells which were not recognized by E2 phage. Fresh spinach leaves, untreated, spiked with *Bacillus anthracis spores* and *E. coli* (100 cfu/25 g spinach), were put into cells pre-enrichment (2.4), with 7 h incubation in LB broth. The resonant frequency shifts of the biosensors, 5 parallel sensors for suspensions derived from each set of samples, were tested (2.5) as negative control. The cells binding on the sensor surface were observed and confirmed by optical microscopy (2.6).

## 3. Results and Discussion

### 3.1. Phage Immobilization and Surface Coverage

**Figure 1:**
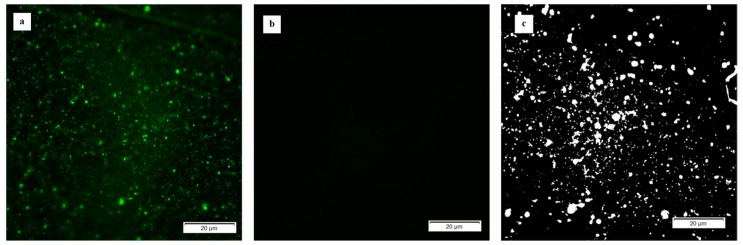
Fluorescence micrographs: (**a**) phage-coated sensor; (**b**) control sensor; and (**c**) binary image.

E2 phage immobilization on the sensor surfaces was confirmed by fluorescent microscopy. Figure 1 shows typical fluorescent micrographs of (a) a phage-coated sensor and (b) a control sensor. The fluorescent pixels shown in Figure 1a are the indication and confirmation of fluorescent-labeled E2 phage adhering to the ME biosensor platform. Based on the fluorescent micrographs (a) and (b) in Figure 1, a binary (black and white) image (c) was made by gray-scale conversion, background subtraction and thresholding. The micrograph of the control sensor was used for background subtraction. The surface phage coverage was then calculated from Figure 1c to be 12.5%, resulting from the exposure to a phage concentration of 2.6 × 10^11^ vir/mL. 

### 3.2. Performance of the Surface Blocking Reagents

**Figure 2:**
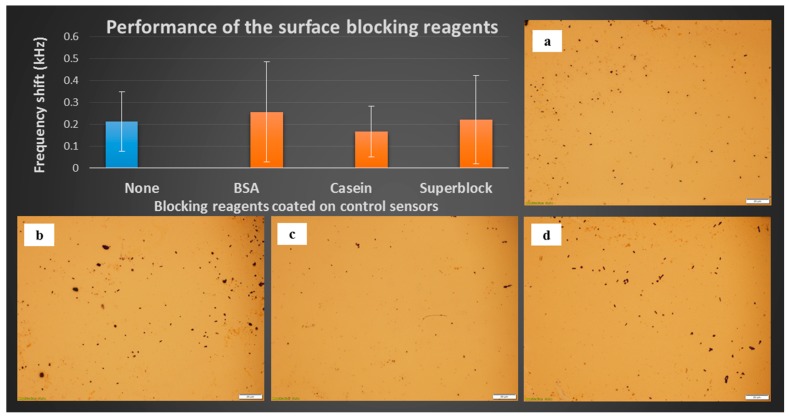
Resonant frequency shifts and sensor surface micrographs (scaleplate 20 µm) after 30 min exposure to 5 × 10^8^ cfu/mL *S.* Typhimurium on bare ME sensor platforms coated with the following blocking agents: (**a**) None; (**b**) BSA; (**c**) Casein; and (**d**) Superblock.

The performance of the blocking reagents was compared using optical microscopy and frequency shift measurements. As can be seen in Figure 2, the frequency shifts and optical micrographs indicate that there was no significant difference among the sensors, with or without the use of blocking reagents. Non-specific binding occurred only to a small degree even for the bare unblocked sensor (Figure 2a). 

The first possible reason is that the phage binding limited the space for the blocking reagents on the surface. The second is that the physical adsorption on the bare gold surface was disturbed by the vibration, while the measurement sensors performed anticipative cell binding.

Therefore, the bare unblocked ME sensor platforms were used as the control sensors for the remainder of this study with little non-specific binding. Shortening the procedure contributes to both cost- and time-effectiveness.

### 3.3. Lactose Broth vs. LB Broth

**Figure 3:**
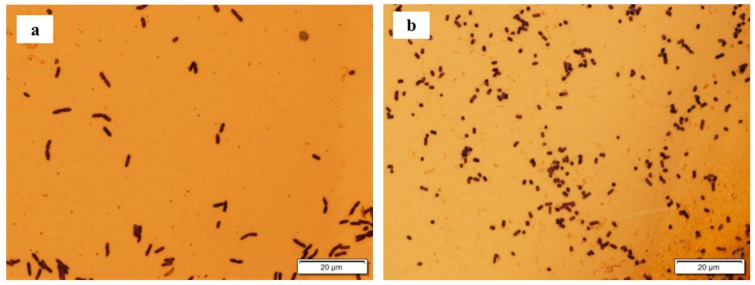
Micrographs of biosensor surfaces after exposure to the suspensions obtained after spinach leaves were incubated in (**a**) lactose broth; and (**b**) LB broth.

Figure 3 shows phage-coated ME biosensors exposed to *Salmonella* suspensions that were derived from incubations of spinach leaves in (a) lactose and (b) LB pre-enrichment broths. Suspensions obtained using LB broth showed a greater quantity of *Salmonella* binding on the ME biosensor surface (Figure 3b) than those obtained using lactose broth (Figure 3a). *Bacillus* cells that were pre-existing on the spinach leaves were observed to non-specifically bind to the biosensor surface for suspensions obtained using lactose broth (Figure 3a). 

### 3.4. Frequency Shifts as a Function of Incubation Time

**Figure 4:**
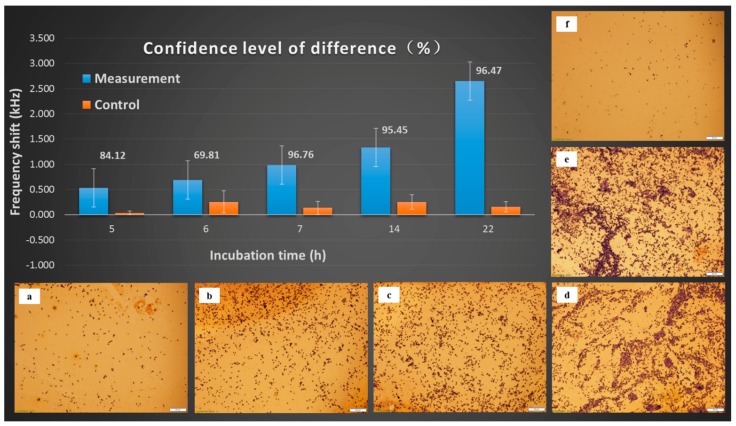
Resonant frequency shifts and sensor surface micrographs (scaleplate 20 µm) for measurement and control sensors placed in the *Salmonella* suspensions after different incubation times in LB broth: (**a**) measurement, 5 h; (**b**) measurement, 6 h; (**c**) measurement, 7 h; (**d**) measurement, 14 h; (**e**) measurement, 22 h; (**f**) control.

Figure 4 displays the frequency shifts and optical micrographs of biosensor surfaces that were exposed to *S.* Typhimurium suspensions derived from the incubation of spinach leaves in LB broth for various lengths of time. The plotted data points are the arithmetic mean values of the frequency shifts with the error bars representing the standard deviations. The numerical percentage values shown are the confidence levels of the difference between the measurement and control sensors determined by a one-tail unpaired Student’s *t*-test.

As can be seen in Figure 4, the frequency shifts of the measurement sensors increase with incubation time while those of the control sensors are much smaller and independent of the incubation time. The optical micrographs of the sensor surfaces corroborate that the frequency shifts correspond to the quantity of cells bound to the biosensor. The control sensors showed much smaller frequency shifts and little nonspecific binding. The frequency shifts of the measurement and control sensors showed a statistically significant difference at 7 h of incubation, with a confidence level of difference higher than 95% (*p* < 0.05).

### 3.5. Negative Control

**Figure 5:**
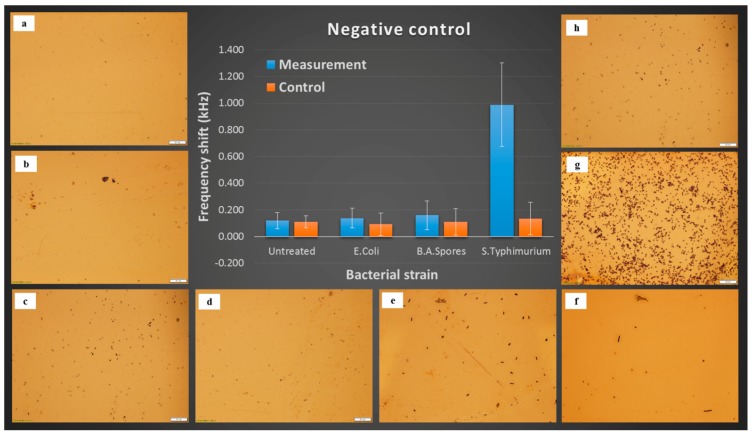
Resonant frequency shifts and sensor surface micrographs (scaleplate 20 µm) for Negative control, 7 h incubation in LB broth: (**a**) untreated, measurement; (**b**) untreated, control; (**c**) *E. coli*, measurement; (**d**) *E. coli*, control; (**e**) *B. A. spores*, measurement; (**f**) *B. A. spores*, control; (**g**) *S.* Typhimurium, measurement; (**h**) *S.* Typhimurium, control.

Figure 5 displays the negative control of the ME biosensor method. Compared to *S.* Typhimurium resulting in a notable signal, the untreated and the masking bacterial cells give weak and stable signals. The optical micrographs confirmed that frequency shifts were caused by non-specific binding.

## 4. Conclusions

An ME biosensor method for *S.* Typhimurium detection on fresh spinach leaves was demonstrated. Following the FDA’s BAM, fresh spinach leaves were spiked with *S.* Typhimurium of known concentration (100 cfu/25 g), and incubated in LB broth in this study. Large shifts in the resonant frequency of the measurement sensors were observed, while the responses of the control sensors were negligible. Optical micrographs verified that the frequency shifts of the measurement sensors were consistent with the amount of *S.* Typhimurium cells attached to the sensor surfaces. Multiple measurement and control sensors frequency shifts were statistically compared and evaluated using a Student’s *t*-test, and the minimum incubation time required for detection was found to be 7 h with a 95% confidence level of difference. This detection time is more than three times faster than the FDA’s current BAM methods (24–26 h). Therefore, the ME biosensor method, for both partial and full detection, was demonstrated to be a robust and competitive method for foodborne pathogens on fresh products.
